# Urbanization threaten the pollination of *Gentiana dahurica*

**DOI:** 10.1038/s41598-018-36773-7

**Published:** 2019-01-24

**Authors:** Qin-zheng Hou, Xia Pang, Yu-pei Wang, Kun Sun, Ling-yun Jia, Shi-hu Zhang, Qiao-xia Li

**Affiliations:** 1 College of Life Science, Northwest Normal University, Lanzhou, Gansu 730030 China; 2 Institute of Modern Physics, Chinese Academy of Sciences, Lanzhou, Gansu 730000 China

## Abstract

With rapid spread of the urbanization, many environmental factors, such as climate, soil pH and nutrients have been changed. However, the plant pollination affected by urbanization was seldom conducted. Here, we studied the flower visitation rates, seed production, pollen limitation and flower morphological characters of *Gentiana dahurica* at 3 populations along an urban-peri-urban gradient around Xi’ning over 4 consecutive years, aiming to test the effects of urbanization on plant pollination service. Our results showed that the pollinator visit frequencies, interannual stability of pollinator assemblages and visit frequencies declined with the intensification of urbanization. As urbanization intensified, plant borne more flowers and the flower morphological sizes became “longer” (the length of flowers, filaments and styles were increased, but the width of flowers kept stable at the 3 populations); the flower duration, especially the female phase duration prolonged. The seed-set ratio of *G*. *dahurica* in natural condition decreased and more severe pollen limitation occurred in more urbanized populations. Also, an interannual variation of seed-set ratio and index of pollen limitation (IPL), which related with the variation of pollinator visit frequencies, were found in this study. These results suggest that the pollination service can be threatened by urbanization over a long-time interval for *G*. *dahurica*. This finding highlights the importance of pollinator affections acting on plant pollination system. Additionally, as pollinator assemblages and visit frequencies interannually changed, a long-time scale observation is needed to understand the plant-pollinator relationships.

## Introduction

Pollination is a critical ecological function in natural and managed systems around the world^1^. It is estimated that 85% of angiosperm species depend on animal pollination to maintain the natural plant populations^2^. However, anthropogenic disturbance, especially the urban expansion, has major impacts on local and regional natural environments^3–5^. In recent years, a large number of researches have been devoted to pollinator declines and alteration of pollination services which were threatened by various anthropogenic disturbances, including habitat destruction and the alteration between agricultural land and urbanization^4,6–9^. While numerous studies were conducted in agricultural systems^4,10,11^, relatively few studies investigate the effects of urban land usage on pollinators and pollination services^12,13^.

Many changes have been caused by the rapid urbanization, such as climate changing^14^, soil pH and nutrients variation^15^, and the habitat fragmentation^16^. The differences between urban and rural are likely to create distinctly selective environments, and therefore affect the plants as well as the insects. Pollinators, firstly, are affected by urbanization for the lack of resources^6^ and suitable habitats at the landscape scales^4,5^. Consequently, pollinator populations need to adjust their foraging and nesting behavior to maintain a sufficient net energetic gain in cities. Some previous studies have targeted on the decline of pollinator caused by the alteration and loss of habitat, especially by the increase of urbanization^12,17–19^. The declined pollinators have a strongly negative effect on plant pollination service as it changes the plant-pollinator interactive networks and consequently impact the reproductive success of plant communities resulting from a strong pollen limitation^20^. However, the effects of urban land use on pollinators remain controversial. For instance, the species richness of bee was found to be negatively^17^, positively^21^, or not affected by urban land use^18^. Taking the time scale into consideration, pollinator abundance and plant-pollinator relationships varied temporally according to the changing environment (such as urbanization)^1,2^. A short-term observation of pollinator abundances could not comprehensively understand the urbanization effect on the pollinators. While, to our knowledge, the long-term observation, i.e. year-to-year variation, of the pollinator abundances and plant seed sets affected by urbanization was seldom studied.

Life phenotypic traits are affected by the rapid urbanization^22^. Examples both come from animals, such as crested anoles (*Anolis cristatellus*)^23^ and killifish^24^, and plants, such as *Lepidium virginicum*^25^ and white clover^26^. By planting *Lepidium virginicum* in five urban and nearby rural areas, Yakub and Tiffin concluded that selection in urban environments favors different traits compared with rural and these differences can drive adaptation and shape population structure^25^. However, whether urbanization has ecological effects on floral (or inflorescence) traits are still poorly understood. An important clue for the effect of urbanization on floral traits is through pollinators. As propagative organ, flower morphological characteristics are suitable for pollination. So for entomophilous plants, floral syndrome shaping is strongly correlated with pollinators. Evidence showed that, pollinators, including effective pollinators and suboptimal pollinators, can act as selective forces on the evolution of floral traits^27,28^. Natural selection of pollinators continues to shape the morphology of flowers^29,30^ and the architecture of inflorescences^31^, resulting in changed flower traits, and consequently changed pollination service system^32^. A number of past studies have evaluated pollinator-mediated selection on floral traits in plants, as well as the pollination service system^32–40^. As pollinators are affected by urban land use and the plant populations in urban areas are usually small and strongly isolated leading to a negative effect on the pollen transport, how urbanization affect the floral variation and plant pollination service are still not clear.

We studied the reproductive success, pollen limitation and floral traits of *Gentiana dahurica*, a common species in the northwest China, at 3 subjected populations over an urban–peri-urban gradient in Xi’ning over 4 consecutive years. In terms of the distance, we primarily defined this gradient as the distance to metropolis. More specifically, we collected related data on pollinator assemblages and flower visitation rates over four continuous years. The aims of this study are to investigate: (i) the relationship between pollinator visitation rates and urbanization along the year to year changing (ii) floral morphological characters of *G*. *dahurica* influenced by urbanization, and (iii) pollen limitation and seed-set ratio affected by urbanization.

## Material and Methods

### Study sites and species

During 2010–2013, we carried out our studies at 3 natural populations: site PJ was situated at Pengjiazhai (lat. 36°37′85′′, long. 101°43′6′′, alt. 2400), which locates at the edge of the metropolis (2.75 km to the center of Xi’ning); site XZ was situated at Xiaozhai (lat. 36°37′18′′, long. 101°35′14′′, alt. 2600), 9.5 km to the center of Xi’ning city; site DT was situated at Datong (lat. 36°47′1′′, long. 101°49′31′′, alt. 2330), 17.5 km to the center of Xi’ning (Supplementary Table S1).

*G*. *dahurica*, according to Ho and Liu^41^, has a wild distribution in the northwest and northeast of China. *G*. *dahurica* is an herbaceous perennial plant with a basal vegetative rosette and several stems with the height up to 5–20 cm. One stem, means an inflorescence, can bear several flowers which are not crowded and clustered. The individual flower has an erect, funnel-shaped corolla comprising of five connate petals. The color of the corolla is blue, and it presents to be white at the base of the corolla. There are many spots that distribute from top to bottom of the corolla, which may attract pollinators. Inside the corolla, several clear white parallel strips may guide pollinators to the nectar. The androecium is composed of five separate filaments and five separate anthers. And the gynoecium is composed of a single bicarpellate ovary with parietal placentation bearing numerous lines of ovules.

### Pollinator observation

In the full anthesis phase, we monitored all the pollinators of *G*. *dahurica* on the sunny days at 3 populations for at least 5 days respectively each year (more than 30 h/population/year). The total observation time was more than 380 hours. The observation methods described by Duan *et al*. were used to record behavior and visiting frequencies of each visitor species in the field^42^. We observed 5 plants in each population simultaneously and counted every floral visit during a 1-h period between 9:00 a.m. and 6:00 p.m. on sunny days. Visitors were identified in Institute of Zoology, the Chinese Academy of Sciences. Because corolla lobes of *G*. *dahurica* closed at night, we did not carry out observations of floral visitors at night.

Coefficient of Variation (CV) was used to evaluate the interannual variability of pollinator visit frequencies among the 4 continuous years in each population. To calculate CV in an interest population, the total visit frequencies of different pollinators in a subject year were summed. In this case, we did not regard to the different pollinator visit efficiencies. Similarly, we used the total visit frequencies to explore the difference of pollinator visit frequencies among the 3 populations. We calculated between-year similarity in observed pollinators using the Jaccard index, proposed by Petanidou *et al*.^43^, i.e. the number of pollinator species present in both years divided by the total number of pollinator species recorded across the 2 years.

### Floral traits

In full-bloom stage of *G*. *dahurica*, we selected 100–150 fully opening flowers on different plants in each population to test the flower sizes at the 3 populations. To avoid the position effect as much as possible, we did not choose the terminal flowers because usually the terminal flowers were withered in the full anthesis phase (all the flowers and buds were selected in the same way below). By using vernier caliper, we measured the length of flowers (LF, length from the top to the bottom of the flower), width of flowers (WF, flower diameter), length of filaments (LFi, average length from the top to the bottom of the 5 filaments) and length of style (LS, length from the top to the bottom of the style) of *G*. *dahurica* at all the 3 populations. Also, we randomly selected 100–150 plants and counted the flower numbers per plant, including buds and fruits. To test the sex allocation changes of *G*. *dahurica* among the 3 populations, 30 buds on different plants of *G*. *dahurica* in each population were selected randomly. Then the pollen numbers (PN) and ovule numbers (ON) were counted. The pollen/ovule ratio (P/O) were calculated as P/O = pollen numbers in all five anthers/ovule numbers. The normality of floral traits was tested using 1-K-S (one sample Komogorov-Smirnov), and then the data was compared using one-way ANOVAs (with Tukey’s multiple contrasts) separately for each flower trait.

The flower development and lifespan were investigated in the field. We did this at the 3 populations at the same time because the phenologies were almost the same (although we didn’t record the phenology data). We recorded 10 flowers on different plants at each population individually. The flowers were marked in the bud stage. Following the methods described by Duan *et al*.^42^, the pollen and stigma presentation were monitored during a 2-h period between 9:00 a.m. and 6:00 p.m. until the flowers withered. The pollen presentation (male phase) were recorded as time span from the commencement of flower opening to initial stigma lobe opening, and the stigma presentation (female phase) were recorded as time span from the initial stigma lobe opening to permanent closure. The normality of data was tested using 1-K-S, and then one-way ANOVAs (with Tukey’s multiple contrasts) were used to test the difference of male and female lifespan among the 3 populations.

### Reproductive success and pollen limitation

*G*. *dahurica* is self-compatible^44^. To test whether autonomous selfing occurs in *G*. *dahurica*, we subjected the flowers of *G*. *dahurica* to the treatments of isolation (flowers were isolated with paper bags before open) in 2011 and 2012 at the 3 populations. 50 buds on different plants were randomly selected and all the anthers were removed before flowers open. Furthermore, to test whether facilitated selfing result from insects occurred, we tagged 50 individual plants at each population in each year. For each tagged plant, 2 individual buds were selected, one was assigned to natural pollination (control), and the other was assigned to remove all the anthers before stigma lobe opening (emasculation). When mature, all fruits from these plants were collected and all the seeds, including mature and abortive seeds, were counted. Seed-set ratios were used to assess the breeding results of each treatment, calculated by mature seeds divided by the total ovules in each ovary. A paired *t* test was used to analyze the difference between the seed-set ratio of control and emasculation.

For every population in each year, we tagged 50 individual plants. For each plant, we tagged 2 buds at the same position on the inflorescences, one bud was assigned to natural pollination (control) and the other to supplemental hand pollination when stigma lobe opened. For the experiment of supplemental hand pollination, the pollen was collected randomly with tweezers from unmarked individuals at a minimum distance of 5 m from the recipient individual. Supplemental hand pollinations were conducted during peak flowering periods and the experiment was conducted until the flower permanent closured. When mature, all seeds were counted, and seed-set ratios were calculated. For each population and year, we calculated an index of pollen limitation^45^ (IPL): IPL = 1 − (Po/Ps), where Po is control seed-set ratio and Ps is the supplemental cross pollination seed-set ratio. To evaluate the interannual variability of IPL and natural seed-set ratio of *G*. *dahurica* among the 4 continuous years at each population, Coefficient of Variation (CV), which is a data dispersion parameter, was used. CV was calculated as CV = (standard deviation/mean) *100%.

The normality of seed set ratio was tested using 1-K-S, the results showed that the seed set ratio was normal distribution (Z = 0.785, *p* = 0.569). Then GLM (General Linear Model) with three-way ANOVAs were used to test the effects of flower treatments (control and supplemental pollination), populations, and years on seed set ratio of *G*. *dahurica*.

### Relationship between pollinator visit frequencies and reproductive success

To explore the relationship between pollinator visit frequencies and reproductive success, we tested the regressive relationships between pollinator visit frequencies with natural seed-set ratio and IPL. The data were calculated as 3 population*4 years = 12. The data of insect visitation were log_10_ (x + 1) transformed to improve normality and homogeneity of variance.

## Results

### Flower pollinators

**Figure 1:**
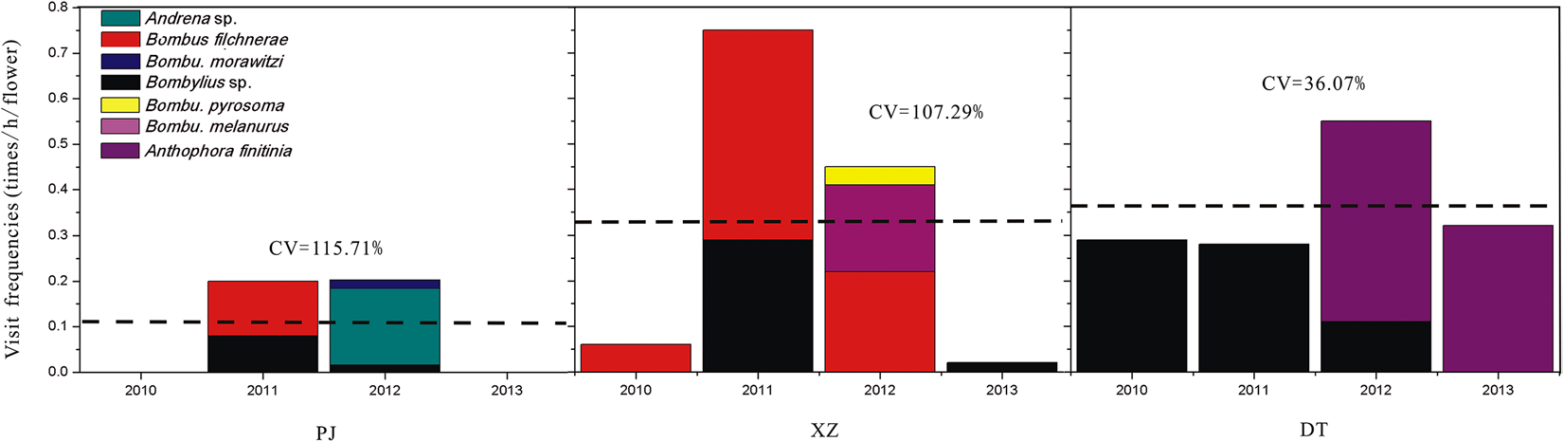
The pollinator assemblages and visited frequencies of *G*. *dahuric* at the 3 populations in 4 years. The visited frequency expressed as flower visit times/h/flower. CV means Coefficient of Variation; the imaginary line means mean value of visited frequencies in each population.

Over the entire 4-year study period, we recorded 7 different pollinator species of *G*. *dahurica*. However, the pollinator assemblages varied significantly in different years and populations. *Bombylius* sp. was commonly seen at population DT in 2010–2012, but not seen in 2013; *Bombylius* sp. was also seen at population PJ and XZ in 2011; *Anthophora finitinia* was specially seen at population DT in 2012 and 2013; *Bombus filchnerae* was commonly seen at population XZ in the four observed years, as well as population PJ in 2011 and 2012, but never seen at population DT; *Andrena* sp. and *Bombu*. *morawitzi* were specially seen at population PJ in 2012; *Bombu*. *melanurus* was sepecially seen at population XZ in 2012 (Fig. 1).

**Figure 2:**
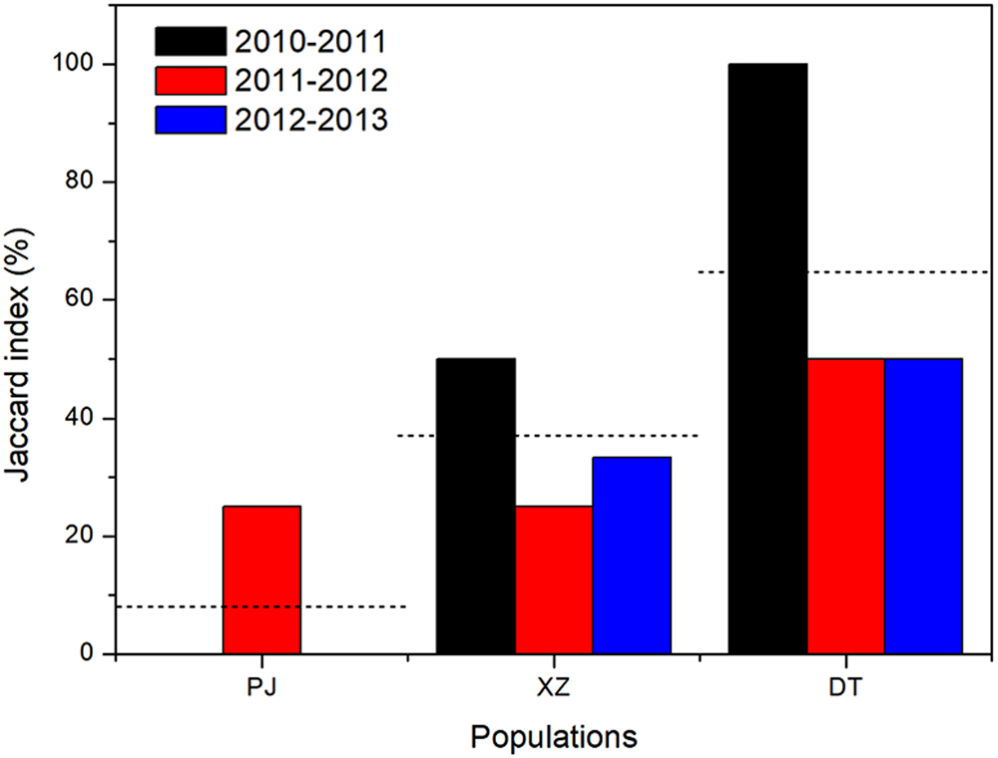
The Jaccard similarity index across 2 years of *G*. *dahurica* in 4 years. The imaginary line means mean value of Jaccard index in each population.

Considering population as fixed factor, the pollinator visit frequencies of *G*. *dahurica* at population XZ and DT (0.32 and 0.36 times/h/flower respectively) were significantly higher than which at population PJ (0.11 times/h/flower) (Fig. 1). The interannual variability of pollinator visit frequencies at population PJ and XZ (CV = 115.71% and 107.29% respectively) were significantly stronger than which at population DT (CV = 36.07%) (Fig. 1). The Jaccard similarity of pollinator communities between any pair of years varied from 0 to 25% (average 8.33%) in population PJ, from 25% to 50% (average 36.11%) in population XZ, and from 50% to 100% (average 66.67%) in population DT (Fig. 2). According to the Jaccard index at the 3 populations, the pollinator component stability cross the 3 populations was DT > XZ > PJ.

### Floral traits

**Figure 3:**
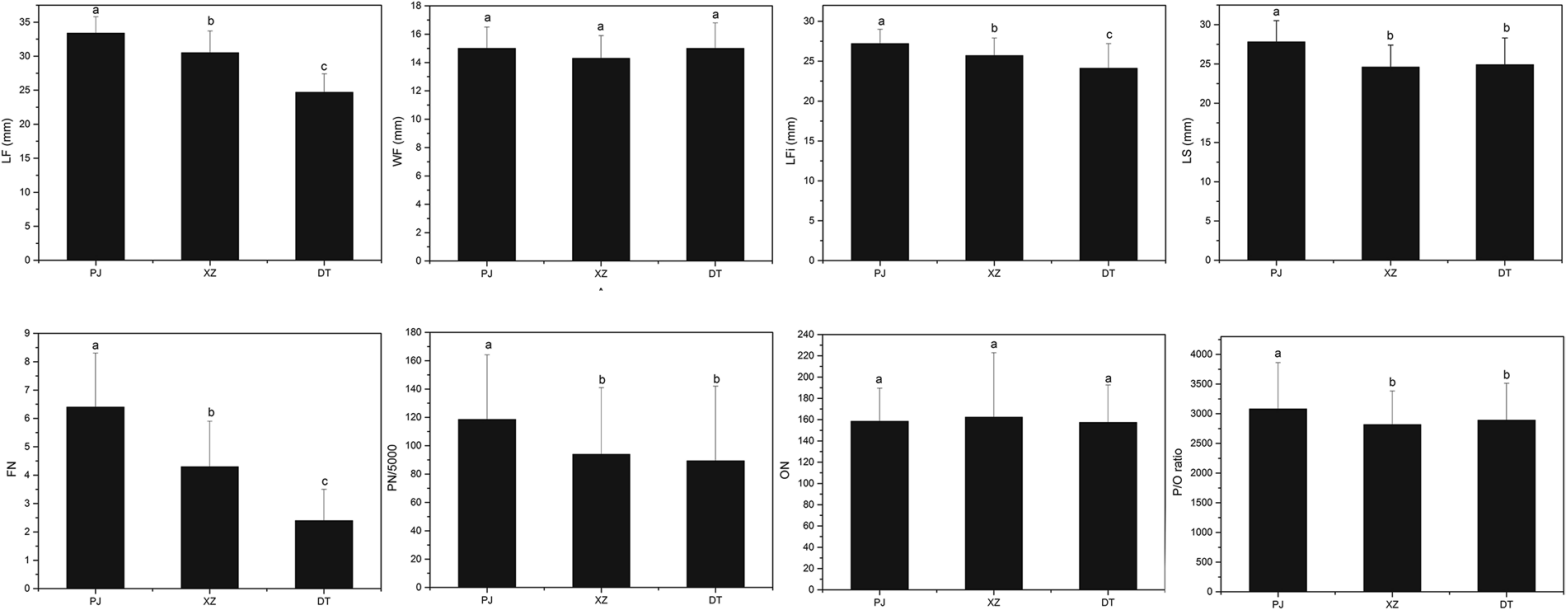
Flower traits of *G*. *dahurica* at the 3 populations. The Different letters in an item means differ significantly at the 0.05 level. LF: length of flowers; WF: width of flowers; LFi: length of filaments; LS: length of styles; FN: flower numbers per single plant; PN: pollen grain numbers; ON: the ovule numbers.

The morphometric traits of *G*. *dahurica* varied significantly at different populations, especially in the length of flowers (LF), length of filaments (LFi), and length of style (LS). *G*. *dahurica* at population PJ had the longest LF and LFi, while it was the converse at site DT. The length of style (LS) at population PJ was longer than the other two populations. For the width of flowers (WF), no significant difference was found among the 3 populations (Fig. 3). As to the flower numbers per single plant (FN), *G*. *dahurica* bear most flowers per plant at site PJ, and least at site DT. The pollen grain numbers (PN) and P/O ratio of *G*. *dahurica* at population PJ were higher than which at the other two populations, but the ovule numbers (ON) showed no significant difference among the 3 populations (Fig. 3).

Usually, the terminal flowers on the inflorescences open first, but the other flowers on the same inflorescence do not follow any order. The flowers of *G*. *dahurica* are protandrous and change from pre-dehiscent to staminate and pistillate. During the life span of a single flower, five stamens occupied the middle of the flower firstly, then they scattered until closed to the inner corolla. At the same time, the pistil elongated, and the stigma occupied the position of middle corolla, then stigma opened, and exudates were released. This phenomenon indicated that herkogamy occurred in *G*. *dahurica*. The floral duration of *G*. *dahurica* was longer at population PJ (5.8 ± 0.4 d) than the other two populations (4.7 ± 0.2 d at population DT and 4.8 ± 0.6 d at XZ respectively). The male phase duration at the 3 populations did not differ significantly, but the female phase duration of population PJ (2.9 ± 0.1 d) was longer than population at DT (2.1 ± 0.1 d) and XZ (2.0 ± 0.0 d) (Supplementary Table S2).

### Reproductive success

**Figure 4:**
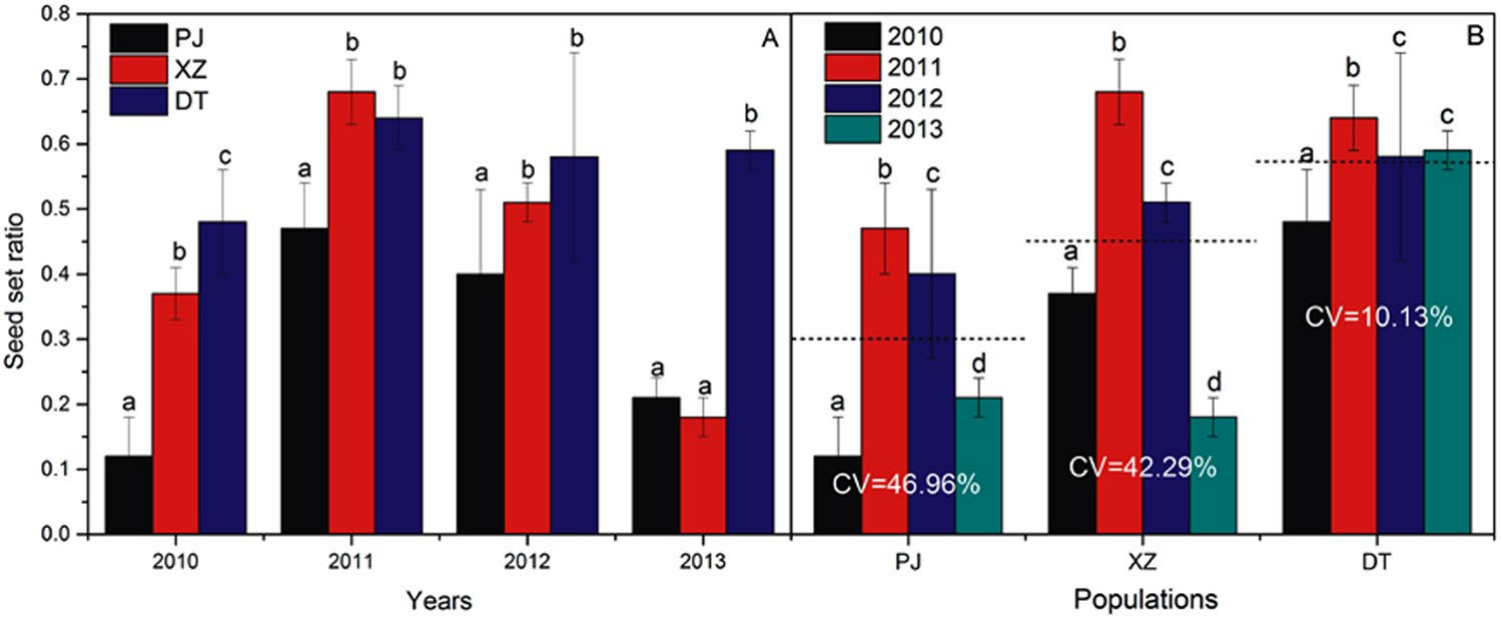
Natural seed-set ratio of *G*. *dahurica* at the 3 populations (**A**) in the 4 years (**B**). Different letters in an item means differ significantly at the 0.05 level. CV means Coefficient of Variation; the imaginary line means mean value of seed-set ratio in each population.

**Figure 5:**
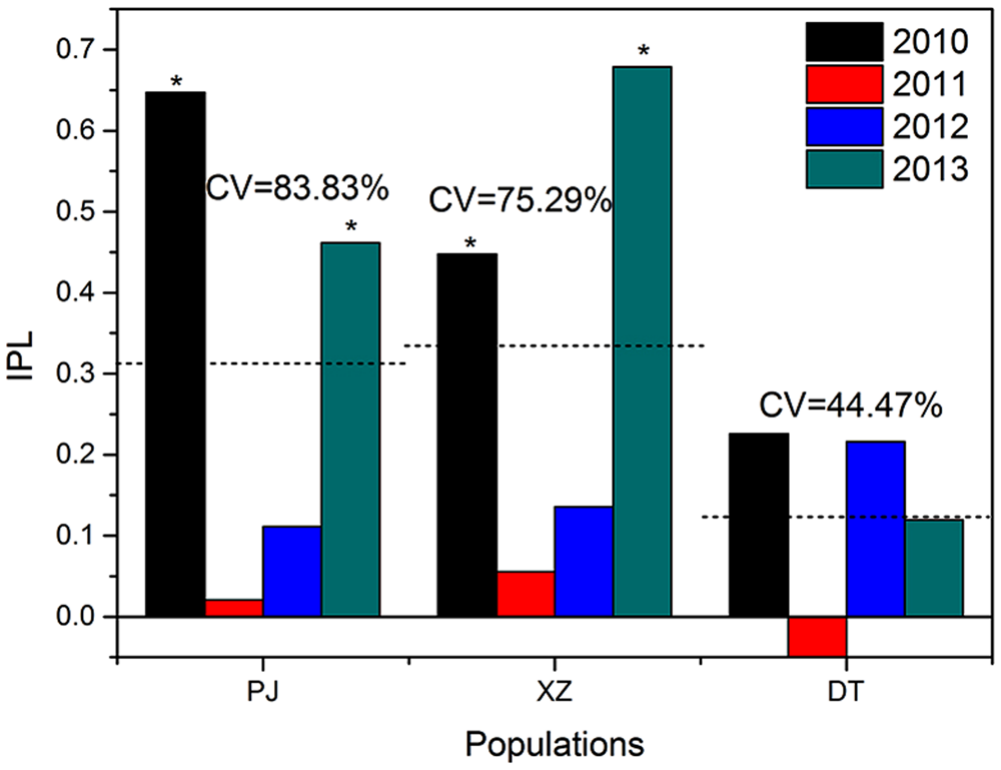
The IPL (index of pollen limitation) of *G*. *dahurica* in the 4 years at each population. *Means a significant effect of the supplemental pollination at the 0.05 level; CV means Coefficient of Variation; the imaginary line means mean value of IPL at each population.

When isolated, none seed was produced over the subjected 2 years, indicating that no autonomous self-pollination occurred in *G*. *dahurica*. There was no significant difference of seed-set ratio between the emasculated flowers and control, indicating that no facilitated selfing occurred in *G*. *dahurica*. The natural seed-set ratio was significantly affected by populations in each year (Table 1; Fig. 4A). Although we did not detect the significant differences between population XZ and DT in 2011, as well as in population PJ and XZ in 2013, a significant difference was detected among the 3 populations when population used as fixed factor, the difference of the seed-set ratio was that population DT > XZ > PJ (Table 1; Fig. 4A). The natural seed-set ratio was also significantly affected by years in each population (Table 1; Fig. 4B), the interannual variability of seed-set ratio at population PJ and XZ (CV = 46.96% and 42.29% respectively) were obviously stronger than which at population DT (CV = 10.13%) (Fig. 4B). Pollen limitation did not occur in population DT in all the 4 consecutive years, as well as in population PJ and XZ in 2011 and 2012, but a significant pollen limitation was detected in population PJ and XZ in 2010 and 2013 (Fig. 5). The IPL at population PJ and XZ did not differ significantly, but significantly higher than which at population DT. Furthermore, the interannual variability of IPL at population PJ and XZ (CV = 83.83% and 75.29% respectively) were also stronger than which at population DT (CV = 44.47%) (Fig. 5).

**Table 1:** Three-Way ANOVA analysis with flower treatment, population, and year as fixed factors for the seed-set ratio of *G*. *dahurica*.

Source	Type III SS	*df*	MS	*F*	*p*
Intercept	8.13	1	8.132	263.52	<0.001
Treatment	0.8	2	0.4	13.33**	<0.001
Population	2.29	2	1.145	38.17**	<0.001
Year	1.79	3	0.597	19.89**	<0.001
Treatment*population	0.19	4	0.049	1.62	0.085
Treatment*year	0.53	6	0.088	2.92**	0.005
Population*year	0.55	6	0.091	3.13**	0.002
Treatment*population*year	0.23	12	0.02	0.65	0.342
Error	9.37	312	0.03		

Significant F values are underlined. SS = sum of squares. **p < 0.01.

### Relationship between pollinator visit frequencies and reproductive success

**Figure 6:**
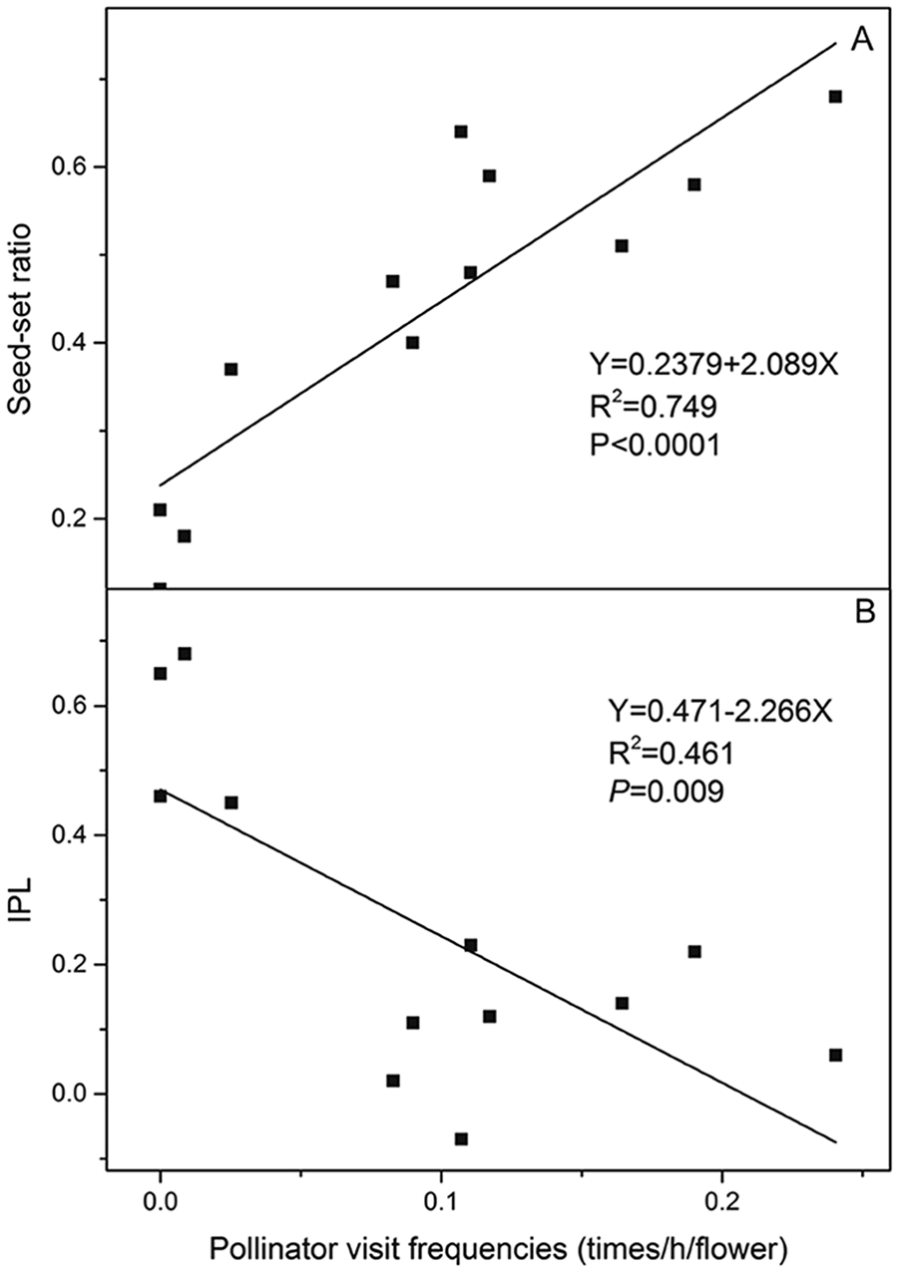
The regression analysis of pollinator visit frequencies with seed-set ratio (**A**) and IPL (**B**) of *G*. *dahurica*.

According to the regression analysis of the 12 sets of data, we found that the natural seed-set ratio of *G*. *dahurica* had a significantly positive correlation with pollinator visit frequencies (Fig. 6A). In contrast, the IPL had a significantly negative correlation with pollinator visit frequencies (Fig. 6B). Our data indicated that the pollinator activities could influence the natural see-set ratio and IPL along with space-time changing.

## Discussion

Pollinator richness, which is a key factor to the plant reproductive success, has been proved by hundreds of experiments that it could be influenced by environmental conditions^46^. The environmental changes at the urban-peri-urban gradient may influence the pollinator richness, but the effects were proved to be different (increase, decrease, or no influence at all)^17,18,21^. Our results support the point that urbanization might decrease the pollinator richness and activity, since the pollinator diversities and visit frequencies of *G*. *dahurica* decreased along the urban-peri-urban gradient. Importantly, we found that the stability of pollinator visit frequency was weaker as urbanization intensified (Fig. 1). Based on the results, we considered that urbanization might negatively affect pollinators in two ways (pollinator activity and stability). This conclusion was also proved by the Jaccard similarity index across 2 years of *G*. *dahurica* in 4 years, since which were generally declined along with the distance near to the metropolis (Fig. 2). It is hard to know the exact reason for the decline of pollinator activities and stabilities along with the urbanization intensified, since the changed environment resulted from urbanization could act on insects in many ways^47,48^. Accordingly, we consider that a longer time-scale observation was needed to verdict the affection of urbanization on pollinator richness and activity, especially at the population near to metropolis, since which might be interannually different (Figs 1, 2). Interestingly, at the population XZ, which located in a middle distance to metropolis compared to the other populations, the average pollinator visit frequency was high, but highly variable (Fig. 1). Compared the population XZ with PJ (with low and highly volatile pollinator visit frequencies) and DT (with high and lowly volatile pollinator visit frequencies), we speculated that urbanization might progressively influence the pollinator activities, i.e. firstly break down the bistable plant-pollinator relationships, and then reduce the pollinator visit frequencies. Continually, we estimated that the pollinator richness and plant reproductive service would more easily be interrupted along with urbanization going on^49–51^.

Flower morphological characteristics were suitable for pollination. As to entomophilous plants, pollinators were the principal elements in floral syndrome shaping^28,35^. Our results showed that, as urbanization intensified, plant borne more flowers and the flower size became “longer” (the length of flowers, filaments and styles were increased, but the width of flowers kept stable at the 3 populations) (Fig. 3). Correspondingly, we observed that as urbanization intensified, the pollinator stability and activity decreased (Fig. 1), which result in an effective selective pressure for the urbanized plants. Although “longer” flower sizes would intensify the plant energy consumption, the increased flower morphology sizes and more flower numbers could enhance the floral display and pollinator attraction to ensure reproductive success^28,52,53^. This was confirmed by more pollen numbers, higher P/O ratio and longer floral duration (especially female duration) of *G*. *dahurica* at more urbanization-intensified populations (Fig. 3, Table S2 in Supporting Information). These characters could prolong the flower display time and increase male fitness, which were effective adaptive strategies to the interference habitats^54–56^. Besides affected by pollinator activities and visit frequencies, floral traits often differentiate in association with pollinator functional group shifts both in specialized and generalized pollination plant species^27,28^. In our study, we detected 7 pollinator species and the pollinator assemblages varied among the 3 plant populations, but all these pollinators were bees. Based on the pollinator visit behaviors and pollen transfer modes, the 7 species belonged to one pollinator functional group, which would provide little contribution to the floral trait variation^35^. Furthermore, for the perennials, because reproduction is costly in plants, current failures in seed production might induce the enhanced investments to future reproduction under the hypothesis on the trade-off between current and future reproduction^57^. The present study showed that the seed-set ratio of *G*. *Dahurica* decreased as urbanization intensified (Fig. 4), which would be a trade-off strategy inducing more resource investments on the floral structure, such as more pollen numbers, higher P/O ratio and longer floral duration.

Neither selfing nor facilitated selfing occurred at all the 3 populations, indicating that urbanization did not change the pollination mode of *G*. *dahurica*. As under natural condition, the overall signal was a drop in seed-set ratio at the populations where more closed to metropolis (Fig. 4; Table 1), which was consistent with past studies^58,59^. However, some other studies proved that the seed-set ratio might increase with the urbanization^60–62^. Although antipodal conclusions existed, they concluded that the increase or decrease of seed-set ratio at urban was consistent with pollinator activities. In our study, the decline of seed-set ratio of *G*. *dahurica* followed the decline of pollinator visit frequencies at the 3 populations. Furthermore, we found a stronger fluctuation of seed-set ratio and IPL at the populations where more closed to metropolis (Figs 4, 5). Correspondingly, the pollinator visit frequencies were lower and the volatilities were stronger at more intensified urbanization populations. This was confirmed by the regression analysis of pollinator visit frequencies with seed-set ratios and IPL (Fig. 6). These results support the idea that urbanization could decrease plant seed production and seed production stability by pollinator actions. Nevertheless, the seed production is also dependent on other parameters, including non-uniform pollination, resource limitation, architectural effects^63^ and local environmental conditions^64^. We did not control all environmental conditions and the sensitivity of seed production to local conditions could scramble the influence of urbanization on pollination^59^. Although we proved that pollinators affirmatively affect the plant fecundity, urbanization could also provide a multifactor functioning on plant reproduction. Thus, more experiments needed to be explored the affection of other parameters, as well as their interrelationships, on plant reproduction. On the other hand, from an evolutionary perspective, the plants near to metropolis will take more risks of extinction, especially for the obligatory outcrossing, entomogamous species and endangered species^49,50^.

## Electronic supplementary material

Supplementary Table S1 S2
