# Supplementary material for: Urbanization threaten the pollination of Gentiana dahurica
Source: Sci Rep. 2019 Jan 24;9:583. doi: 10.1038/s41598-018-36773-7 (PMC6345766; doi:10.1038/s41598-018-36773-7)
Supplement: Supplementary file 1 — Supplementary Table S1 S2 [file 41598_2018_36773_MOESM1_ESM.pdf]

Urbanization threaten the pollination of *Gentiana dahurica*

Qin-zheng Hou[1], Xia Pang[1], Yu-pei Wang[2], Kun Sun[1], Ling-yun Jia[1], Shi-hu Zhang[1] and Qiao-xia Li[1]

[1]College of Life Science, Northwest Normal University, Lanzhou, Gansu, 730030, China
[2]Institute of Modern Physics, Chinese Academy of Sciences, Lanzhou, Gansu, 730000, China

*Correspondence and requests for materials should be addressed to Q. Z. H. (email: hou_qzh@163.com).

Supplementary Table S1. The environment conditions of 3 *G. dahurica* populations.

Population	Location	Distance to the center of the city (km)	Land use type	Main accompanying species
PJ	lat. 36°37′85″<br>long. 101°43′6″<br>alt. 2400	2.75	pasture	*Poa* L., *Potentilla* L., *Oxytropis* DC.
XZ	lat. 36°37′18″<br>long. 101°35′14″<br>alt. 2600	9.5	pasture	*Carex* L., *Poa* L., *Salix* L.
DT	lat. 36°47′1″<br>long. 101°49′31″<br>alt. 2330	17.5	pasture	*Carex* L., *Anaphalis* DC., *Poa* L.,

Supplementary Table S2. One-way ANOVA analysis with flower total duration, male phase duration and female phase duration of *G. dahurica* among 3 populations.

Source		Type III SS	df	MS	*F*	*P*
Total duration	Between groups	22.067	2	11.033	6.043	0.007
	Within groups	49.300	27	1.826		
	Total	71.367	29			
Male phase duration	Between groups	4.867	2	2.433	3.318	0.057
	Within groups	19.800	27	0.733		
	Total	24.667	29			
Female phase duration	Between groups	25.800	2	12.900	9.313	0.001
	Within groups	37.400	27	1.385		
	Total	63.200	29			
